# Integrated Phenotypic, Proteomic (MALDI-TOF MS), and Genomic (WGS) Investigation of a Prolonged Hospital Outbreak of *Pseudomonas aeruginosa* with High Biofilm-Forming Capacity

**DOI:** 10.3390/antibiotics15030257

**Published:** 2026-03-02

**Authors:** Sandra Pamela Cangui-Panchi, Danny Santiago Cangui-Panchi, Verónica E. Palacios, Erika Becerra, Ana L. Santamaría, Diana Muñoz, Jorge Reyes-Chacón, António Machado, Daniel Garzon-Chavez

**Affiliations:** 1 Laboratorio de Bacteriología, Instituto de Microbiología, Colegio de Ciencias Biológicas y Ambientales (COCIBA), Universidad San Francisco de Quito (USFQ), Quito 170901, Ecuador; pcanguip@estud.usfq.edu.ec; 2 Asociación Científica de Estudiantes de Medicina, Unidad Académica de Salud y Bienestar, Universidad Católica de Cuenca, Cuenca 010101, Ecuador; danny.cangui.39@est.ucacue.edu.ec; 3 Hospital del Instituto Ecuatoriano de Seguridad Social (IESS) Quito-Sur, Quito 170111, Ecuador; veronica.palacios@iess.gob.ec (V.E.P.); eri.jacke90@gmail.com (E.B.); asantamariaor@gmail.com (A.L.S.); diana.munozloja@iess.gob.ec (D.M.); jorgereyes83@gmail.com (J.R.-C.); 4 Facultad de Ciencias Químicas, Universidad Central del Ecuador, Quito 170521, Ecuador; 5 Departamento de Biologia, Faculdade de Ciências e Tecnologia, Universidade dos Açores (UAc), R. Mãe de Deus 13A, 9500-321 Ponta Delgada, Portugal; 6 CIBIO, Centro de Investigação em Biodiversidade e Recursos Genéticos, InBIO Laboratório Associado, BIOPOLIS Programa em Genómica, Biodiversidade e Planeamento, Universidade dos Açores, Rua da Mãe de Deus, 9500-321 Ponta Delgada, Portugal; 7 Colegio de Ciencias de la Salud, Universidad San Francisco de Quito (USFQ), Quito 170901, Ecuador

**Keywords:** outbreak, *Pseudomonas aeruginosa*, biofilm, phenotyping, proteomics, genomics, MALDI-TOF MS, WGS

## Abstract

**Background/Objectives**: Hospital outbreaks of *Pseudomonas aeruginosa* are difficult to control due to the pathogen’s extensive repertoire, including its ability to form biofilms, adapt and persist in diverse environments, and develop multidrug resistance, all of which contribute to prolonged outbreaks. This study integrates the phenotypic, proteomic, and genomic characterization of a nosocomial outbreak comprising 38 clinical isolates and one environmental isolate recovered from the intensive care unit (ICU) of Hospital IESS Quito Sur. **Methods**: Clinical data were collected, antimicrobial susceptibility was assessed by minimum inhibitory concentration (MIC), carbapenemase genes were detected by multiple PCR and immunochromatographic assays, and the biofilm formation index (BFI) was determined. In addition, matrix-assisted laser desorption/ionization time-of-flight mass spectrometry (MALDI-TOF MS) was used for species identification and clustering based on spectral similarity. Twelve representative isolates underwent whole genome sequencing (WGS) to characterize the resistome and virulome and to compare phylogenetic relationships with proteomic clustering defined by MALDI Biotyper Compass Explorer software. **Results**: All isolates were identified as *P. aeruginosa*, and phenotypic antimicrobial susceptibility classified most isolates as multidrug resistant, including 32 CRPA strains. The *bla*_VIM_ gene was detected in 22 isolates, while BFI analysis showed that all isolates formed moderate to strong biofilms. Genomic analysis revealed that most isolates belonged to ST111 and ST253, and both conserved and heterogeneous resistome and virulome profiles, with a broad distribution of determinants related to biofilm formation, stress tolerance, and persistence. Comparison between MALDI-TOF MS and WGS showed predominant concordance in clustering, mainly within subclusters but disagreement at the cluster level. **Conclusions**: The detection of carbapenemases, biofilm-forming ability, and virulence determinants associated with prolonged persistence highlights the need for integrated molecular tools, such as MALDI-TOF MS with MALDI Biotyper Compass Explorer software, to support epidemiological surveillance and to inform strategies aimed at mitigating prolonged hospital outbreaks caused by *P. aeruginosa*.

## 1. Introduction

Healthcare-associated infections (HAIs) are a global health problem affecting approximately 30% of patients in intensive care, and one of the most prevalent nosocomial species is *Pseudomonas aeruginosa*, which causes approximately 7.9% of HAIs, with a 30-day mortality rate in complicated cases, such as bacteremia [1,2]. In the hospital setting, this pathogen is generally transmitted through indirect contact and mechanical ventilation and causes pneumonia, wound infections, urinary tract infections, catheter-associated infections, bacteremia, sepsis, and septic shock [3,4,5,6,7]. The high incidence of HAIs is mainly due to this microorganism’s ability to colonize, persist, and adapt to the hospital environment by contaminating wet surfaces, medical equipment, and invasive devices through biofilm formation [7,8,9]. Moreover, phenotypic diversity has been described between clinical and environmental strains of *P. aeruginosa* (including characteristics related to biofilm formation and stress adaptation), supporting the survival of the pathogen in humid hospital environments as potential reservoirs during outbreaks [10,11,12]. In addition, the widespread multidrug resistance to antimicrobials and biocides reported in *P. aeruginosa* contributes to prolonged colonization and difficult eradication, which can lead to hospital outbreaks [13,14,15,16,17].

Several prolonged nosocomial outbreaks caused by *P. aeruginosa* have been reported, especially strains that produce carbapenemases such as Verona integron-encoded metallo-β-lactamase (VIM), due to the environmental persistence of the pathogen, colonization of hospitalized patients, contamination of sinks, drains, and other moist surfaces, and the spread of resistant and biofilm-forming lineages [3,18,19,20,21,22]. Therefore, outbreak control must be implemented in a timely manner due to the high clinical severity, the risk of continued transmission and spread to other hospital areas, the potential for genetic spread, and the emergence of multidrug resistance that can occur in prolonged episodes of *P. aeruginosa* colonization, especially in carbapenem-resistant strains [9,15,23,24].

For timely outbreak control, molecular typing tools capable of distinguishing between clonality and multiple lineages are necessary, as well as identifying possible transmission routes and informing immediate infection control measures [25,26]. The characterization of the set of clinical symptoms and phenotypic profiles represents the first step in outbreak investigation and control, but it has limitations in inferring the relationship between isolates, which is why molecular typing techniques must be considered [26,27,28]. In this context, matrix-assisted laser desorption/ionization time-of-flight mass spectrometry (MALDI-TOF MS) has become an innovative tool that allows isolates to be identified rapidly and cost-effectively. In addition, the development of software has made it possible to analyze spectral similarity by constructing dendrograms, principal component analyses (PCAs), and pseudogels, contributing to the determination of preliminary clustering of outbreak-related isolates, which even allows for rapid screening and real-time epidemiological surveillance at a lower cost [29,30,31,32]. On the other hand, whole genome sequencing (WGS) is a reference tool used to support outbreak control, including high-resolution phylogenetic inference and detailed characterization of resistance and virulence determinants [25,33]. However, its routine implementation is conditioned by costs, infrastructure, personnel, and complex workflows, highlighting the need for complementary and more accessible approaches [34,35].

Together, MALDI-TOF MS and WGS are widely used tools in outbreak analysis, and the results of these techniques have previously been compared with clinical data associated with isolates and phenotypes. Therefore, in this study, we characterized a hospital outbreak of *P. aeruginosa* by integrating clinical data with phenotypic, proteomic, and genomic analyses. By combining antimicrobial susceptibility testing, biofilm assessment, MALDI-TOF MS-based clustering, and WGS, we aimed to describe the resistance, virulence, and clustering relationships among 38 clinical isolates and one environmental isolate recovered from an ICU sink, and to evaluate the complementarity of proteomic and genomic approaches for outbreak investigation.

## 2. Results

### 2.1. P. aeruginosa Isolates and Clinical-Epidemiological Context

A total of 39 *P. aeruginosa* isolates were included in this study, comprising 38 clinical strains and one environmental isolate recovered from a sink in this ICU (PA_35). Thirty-eight clinical strains of *P. aeruginosa* causing nosocomial infections were isolated from Hospital IESS Quito Sur (Quito, Ecuador), from different specimens, including tracheal aspirate (*n* = 18), blood culture (*n* = 13), urine (*n* = 4), bronchoalveolar lavage (BAL) (*n* = 1), sputum (*n* = 1), and central venous catheter (CVC) (*n* = 1), collected between 27 September 2021, and 24 February 2023, from patients hospitalized in the intensive care unit (ICU) ranging in age from 1 to 85 years, with a median age of 62.5 years, 30 isolates were obtained from male patients and 8 from female patients. The clinical diagnoses associated with *P. aeruginosa* isolation included hospital-acquired pneumonia (HAP) (*n* = 19), bacteremia (*n* = 7), septic shock (*n* = 6), urinary tract infection (UTI) (*n* = 4), urosepsis (*n* = 1), and sepsis (*n* = 1) (see Table 1).

### 2.2. Phenotypic Susceptibility Profile and Molecular Detection of Carbapenemases

Phenotypic antimicrobial susceptibility testing was performed exclusively on the 38 clinical isolates, as the environmental isolate (PA_35) was not processed through the hospital routine AST workflow. The 38 clinical isolates showed high resistance to commonly used antibiotics such as ciprofloxacin (84.2%), cefepime (81.6%), ceftazidime (78.9%), gentamicin (55.3%), and piperacillin-tazobactam (44.7%). The lowest resistance rate was to amikacin (15.8%). All clinical isolates were tested for meropenem, revealing 32 resistant isolates (84.2%), which were therefore classified as carbapenem-resistant *Pseudomonas aeruginosa* (CRPA). Colistin MIC values of 1 and 2 mg/mL predominated. Regarding carbapenemase genes detected using the BioFire^®^ FilmArray^®^ 2.0 multiplex PCR system and NG-TEST^®^ CARBA-5 immunochromatographic assay, *bla*_VIM_ or VIM was identified in 22 isolates (56.4%), including the environmental strain PA_35 (see Table 2).

### 2.3. Biofilm Formation Determined by BFI

**Figure 1:**
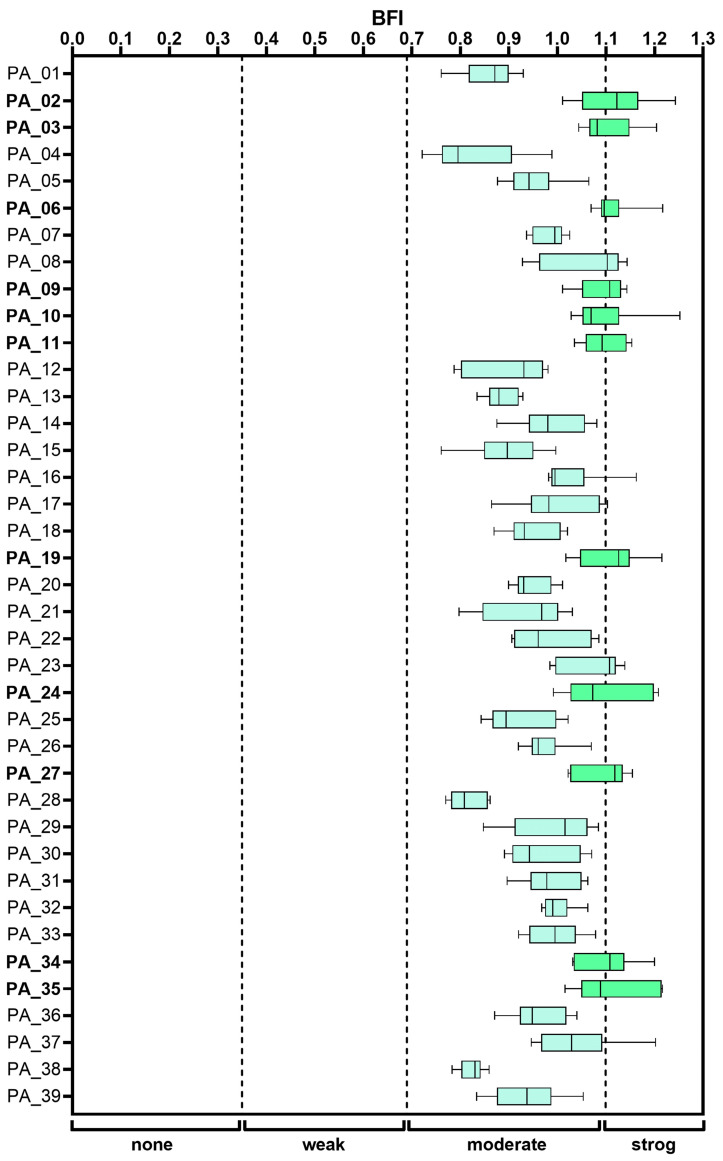
Biofilm formation index (BFI) of *P. aeruginosa* isolates. Boxes represent the median and interquartile range, with whiskers indicating the minimum and maximum values across replicates. Cut-off points divide the categories into none, weak, moderate, and strong biofilm production. Darker green boxes indicate strong biofilm producers, whereas lighter green boxes indicate moderate producers.

The capacity to form biofilms was evaluated in the 39 isolates, and according to the BFI obtained, all isolates were classified as moderate or strong biofilm formers (see Figure 1). Eleven isolates were classified as strong biofilm producers, including the central venous catheter (CVC)-associated isolate PA_34, with a mean BFI of 1.10 ± 0.06, and the environmental isolate from the sink, PA_35, with a mean BFI of 1.11 ± 0.08 (see Supplementary Table S1). Notably, no isolate was classified as a weak or non-biofilm former.

After determining that all *P. aeruginosa* isolates have a high capacity for biofilm formation and are also multidrug-resistant strains recovered from the same area of the hospital, the pattern could correspond to a prolonged nosocomial outbreak, so genomic analysis was performed.

### 2.4. Genomic Characterization of P. aeruginosa Isolates by WGS

Twelve representative isolates of *P. aeruginosa* (including the environmental isolate PA_35) were selected for WGS, and genomic assemblies with adequate continuity were obtained, with no ambiguous bases detected, and taxonomic classification confirmed their assignment to *Pseudomonas aeruginosa* (assembly metrics, coverage, and completeness are shown in Supplementary Table S2).

**Figure 2:**
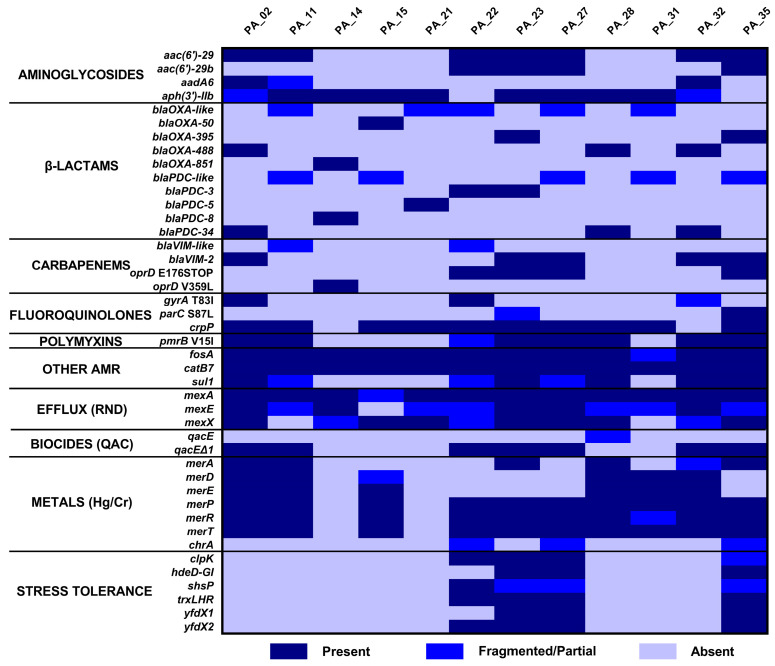
Resistome profile of *P. aeruginosa* isolates obtained by WGS.

Resistome analysis revealed heterogeneous resistance profiles among isolates, based on gene presence, and identified determinants associated with resistance to multiple classes of antimicrobials (see Figure 2) consistent with the phenotype. All isolates previously detected with *bla*_VIM_ were confirmed by genomic analysis. A subset of isolates presented mutations in *oprD* compatible with mechanisms contributing to carbapenem resistance. A high prevalence of genes widely distributed in *P. aeruginosa*, such as *fosA*, *crpP*, and genes encoding RND efflux systems, was observed. In addition, determinants associated with tolerance to biocins, metals, and stress were detected in both the environmental isolate (PA_35) and clinical isolates, consistent with adaptation to hospital environmental pressures and the outcome of a prolonged outbreak with this common environmental reservoir that may have generated a sustained bacterial transmission.

**Figure 3:**
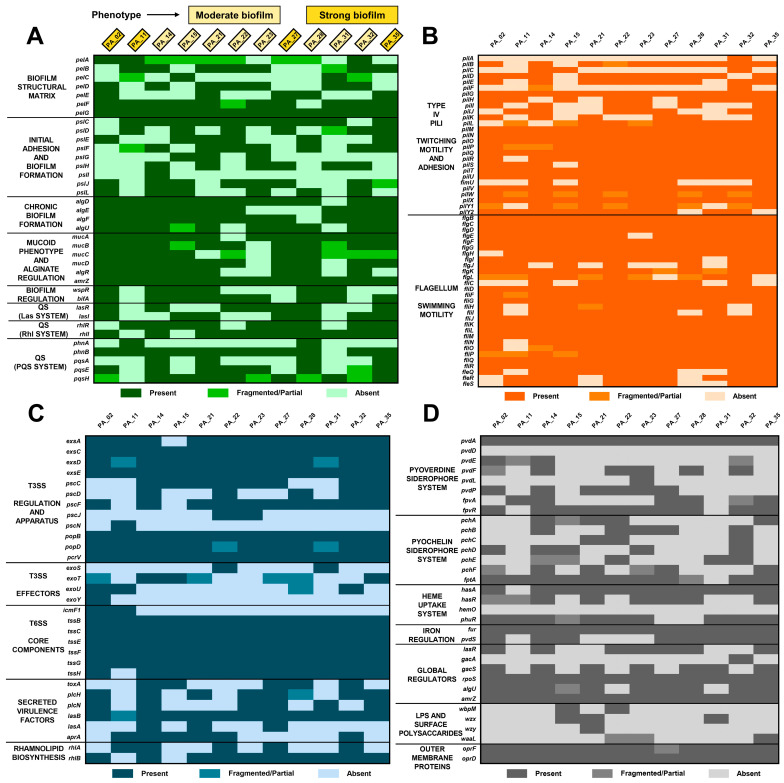
Virulome profile of *P. aeruginosa* isolates obtained by WGS. (**A**) Biofilm and quorum sensing (QS). (**B**) Motility and adhesion. (**C**) Secretion systems, effectors, and toxins. (**D**) Iron acquisition, surface components (LPS/outer membrane), and global regulators.

Virulome analysis showed a high degree of conservation of determinants associated with biofilm formation, particularly those related to alginate biosynthesis (*alg* genes) and mucoid regulation (*muc* genes), which are involved in chronic biofilm formation, consistent with the phenotype. Variability was observed in genes related to the structural matrix (*pel* genes), initial adhesion (*psl* genes), and quorum sensing (QS) systems (*las*, *rhl*, and *pqs* genes) modules, which may reflect differences in virulence regulation and community behavior (see Figure 3A). Determinants associated with motility and adhesion, including type IV pili and flagellar systems, remained largely conserved, with sporadic absence of specific genes (see Figure 3B). Genes encoding the regulatory components of the type III secretion system (T3SS) were mostly present; however, effector genes (*exo*) were absent, whereas the core components of the type VI secretion system (T6SS) were more conserved, while genes encoding secreted virulence factors and rhamnolipid biosynthesis displayed greater heterogeneity (see Figure 3C). Finally, genes involved in iron acquisition and global regulation were largely conserved, whereas components of the pyoverdine system showed heterogeneity, and lipopolysaccharide (LPS) and surface polysaccharide genes exhibited higher levels of absence across isolates (see Figure 3D).

Information on the resistome and virulome gene lists of the sequenced isolates can be found in Supplementary Tables S3 and S4.

Therefore, the presence of genes associated with high biofilm formation capacity, with virulence determinants that favor adhesion, motility, and bacterial transmission, together with mechanisms of persistence and tissue damage, were consistent with the hospital adaptation profile and determined the occurrence of an intra-hospital outbreak in the ICU.

### 2.5. Proteomic Profile of P. aeruginosa Isolates from the Outbreak

The existence of a nosocomial outbreak was confirmed based on the concordance between phenotypic and genotypic findings in *P. aeruginosa* isolates recovered during the same period and in the same hospital environment, mainly due to the shared multidrug resistance to antibiotics and biocides and the high capacity to form biofilms between the environmental isolate and the clinical isolates. Therefore, MALDI-TOF MS was used for the comparative analysis of isolates as an early intervention tool in response to the outbreak.

All outbreak isolates were identified at the species level as *Pseudomonas aeruginosa*, with scores ranging from 2.03 to 2.48. Most isolates were classified as consistency category A (see Supplementary Table S5), corroborating the identity determined at the phenotypic level.

**Figure 4:**
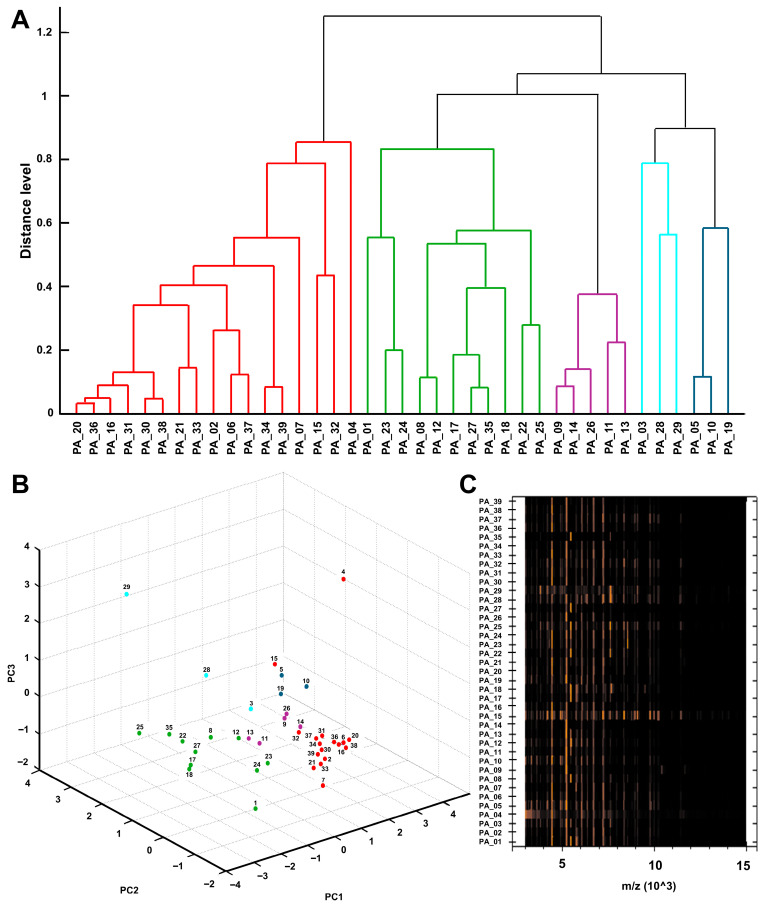
Proteomic profile of *P. aeruginosa* outbreak isolates by MALDI-TOF MS. (**A**) Spectral similarity dendrogram. (**B**) Principal component analysis (PCA) of spectral profiles. (**C**) Pseudogel (gel view) of processed spectra.

Additionally, the proteomic spectral similarity analysis of the outbreak isolates generated a dendrogram with MALDI Biotyper Compass Explorer software that showing an initial hierarchical partition into two major groups. The first group corresponded to the red cluster (*n* = 17) and included the strain isolated from CVC (PA_34), while the second group comprised four main proteomic clusters (*n* = 22), with the green cluster encompassing most isolates, including the strain isolated from the sink (PA_35; *n* = 11) (see Figure 4A). The PCA showed the same initial separation into two groups, corresponding mainly to the red and green clusters. In addition, several isolates located distantly from the main cluster, including those forming the blue and light blue clusters, as well as isolates PA_4 and PA_29, were observed, indicating heterogeneity in the protein profiles of outbreak isolates (see Figure 4B). The normalized pseudo-gel, generated based on mass spectral intensity and enabling visualization of spectral peaks and their consistency among isolates, showed higher signal intensity between 3 and 8 (*m*/*z* 10^3^) and global patterns characterized by conserved vertical bands, indicating recurrent peaks within the 4–6 (*m*/*z* 10^3^) region that may represent the core proteomic profile. Additional variations and repetitive bands were observed within the 6–8 (*m*/*z* 10^3^) range, together with a decrease in signal intensity between 8 and 15 (*m*/*z* 10^3^) (see Figure 4C).

### 2.6. Concordance Between the Proteomic Profile by MALDI-TOF MS and the Phylogeny by WGS in Representative Outbreak Isolates

**Figure 5:**
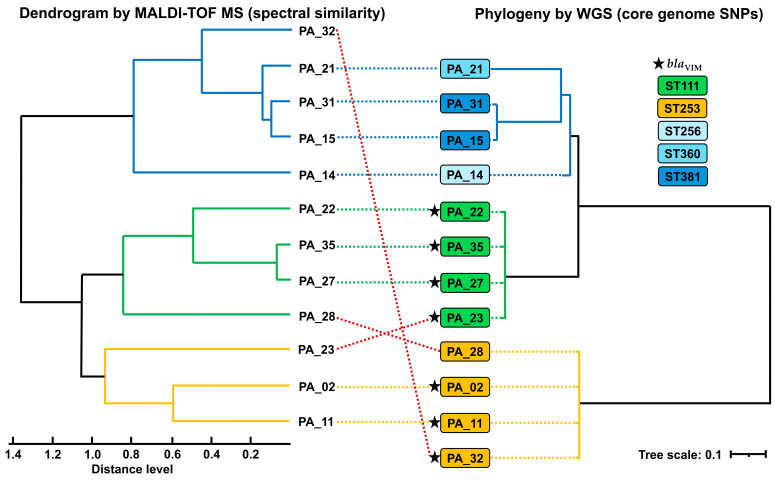
Tanglegram comparing the dendrogram obtained by MALDI-TOF MS (spectral similarity) with the phylogeny inferred by WGS (core genome SNPs). Colored boxes indicate sequence types (STs). Dashed lines connect the same isolates between both trees. Five-pointed stars denote isolates carrying the *bla*_VIM_ gene.

To corroborate the performance of the spectral similarity analysis performed by the MALDI Biotyper Compass Explorer software and document its usefulness as an initial approach to detecting clusters compatible with outbreaks, a tanglegram was generated to contrast the clustering determined by spectral similarity with the phylogeny inferred from core genome single-nucleotide polymorphisms (SNPs), and a predominant concordance (9/12 clustering matches) was observed between the two approaches. Both analyses were organized into two clusters, which were not congruent between methods; however, the subclusters were largely preserved, highlighting closely related isolate pairs across both analytical approaches (PA_31 and PA_15, PA_35 and PA_27, and PA_02 and PA_11) and their consistent sub-grouping within defined clusters. Discrepancies were observed for isolates PA_32, PA_28, and PA_23, which were assigned to different clusters depending on the method used (see Figure 5).

The SNP distances derived from the alignment of the core genome support the structure of the phylogenetic tree, where the environmental isolate from the sink (PA_35) showed greater similarity to the clinical isolates that comprise the same subcluster (PA_23, PA_22, and PA_27 with pairwise SNP distances of 455, 791, and 933, respectively), compared to markedly greater distances (approximately 50,000 SNPs) with isolates from other clusters (see Supplementary Table S6).

In addition, WGS was used to determine the sequence type (ST) of the sequenced isolates, indicated by color in Figure 5. ST111 (*n* = 4) and ST253 (*n* = 4) were predominant, distributed in two distinct clusters in the phylogenetic tree. This grouping structure showed majority agreement with the dendrogram obtained by MALDI Biotyper Compass Explorer software using MALDI-TOF MS spectra. Similarly, the separation of *bla*_VIM_-positive isolates was evident in both WGS and MALDI-TOF MS, with two inconsistencies (PA_28 and PA_32). Overall, these results support the discriminatory power of MALDI Biotyper Compass Explorer software for rapid clustering as an initial screening tool during hospital outbreak investigations.

## 3. Discussion

The isolation in the ICU of the 38 clinical strains of *P. aeruginosa* in different specimens, together with the environmental isolate recovered from a sink, suggests the involvement of this pathogen in a broad clinical spectrum, causing respiratory, invasive, and medical device-associated infections. This pattern is consistent with the ability of *P. aeruginosa* to persist in healthcare settings and adapt to the environment, thereby favoring transmission and recurrence of infections within a framework of clinical severity and epidemiological risk [36,37,38]. Taken together, these findings illustrate how the circulation of *P. aeruginosa* across patients, hospital critical care units, and environmental reservoirs can sustain prolonged outbreaks in critical care settings, highlighting the need to strengthen surveillance and prevention strategies for hospital-acquired infections [28,33,35].

The high rate of resistance to antibiotics used in routine therapy observed among isolates from this outbreak is clinically significant because it limits empirical treatment options in severely ill patients [13,39]. Among these, the high proportion of meropenem-resistant *P. aeruginosa* isolates is particularly relevant, as the World Health Organization (WHO) has designated carbapenem-resistant *P. aeruginosa* (CRPA) as a high-priority pathogen due to its association with high mortality, especially in severe cases of sepsis in the ICU [40,41,42]. Furthermore, the predominant detection of *bla*_VIM_ is consistent with reports identifying VIM as one of the most common metallo-β-lactamases (MBLs) in *P. aeruginosa* in the Americas region [24,43], and with its epidemiological relevance, given its ability to spread via mobile genetic elements and its association with prolonged and difficult-to-control outbreaks [18,21]. The identification of the environmental isolate PA_35 as VIM-producing, together with the predominance of VIM-CRPA among clinical isolates, is consistent with previous outbreak reports linking clinical cases to hospital environmental reservoirs such as sinks, drains, and plumbing systems [19,20,22,23].

In this outbreak, all *P. aeruginosa* isolates were classified as moderate or strong biofilm formers. This phenotype has been associated with increased antimicrobial resistance and reduced efficacy of eradication strategies in hospital-associated infections. The uniform presence of a strong biofilm-forming phenotype across clinical and environmental isolates suggests that biofilm formation constitutes a central trait underpinning persistence and transmission during the outbreak. Other studies have similarly reported high proportions of biofilm-forming clinical isolates, raising concerns regarding their virulence and multidrug resistance potential [15,44]. The strong biofilm phenotype of the environmental isolate PA_35 recovered from the sink is epidemiologically relevant, as it aligns with previously described scenarios in which *P. aeruginosa* adheres to and proliferates on abiotic surfaces such as sinks, drains, and plumbing systems, facilitating environmental persistence and transmission to susceptible patients, thereby promoting outbreaks that are difficult to control, particularly in ICU [20,21,45,46]. Similarly, the strong biofilm-forming capacity observed in the CVC-associated isolate PA_34 is consistent with reports demonstrating the ability of *P. aeruginosa* to colonize medical devices, including intravascular catheters, contributing to bloodstream infections, sepsis and septic shock [6,47,48,49,50,51].

Resistome analysis showed strong concordance between *bla*_VIM_ detection by PCR and WGS, supporting the central role of VIM carbapenemases in the resistance profile of this outbreak. In addition, the identification of alterations in *oprD* suggests the coexistence of multiple mechanisms contributing to carbapenem resistance, a pattern frequently described in CRPA and other high-risk lineages, where mobilome-associated elements contribute to complex resistance profiles [46,52,53]. The heterogeneity observed in aminoglycoside and β-lactamase resistance determinants is consistent with the extensive genomic diversity described for *P. aeruginosa* [54,55,56]. Furthermore, the presence of genes associated with tolerance to biocides, metals, and stress may contribute to the persistence of this pathogen on wet and abiotic surfaces in hospital environments, as described in other outbreak studies [17,33,57,58,59]. Together, the coexistence of multidrug resistance determinants, biofilm-related virulence genes, and stress tolerance mechanisms suggests a genomic background optimized for persistence rather than acute virulence.

The virulome profiles identified in outbreak isolates are consistent with previously reported virulent mechanisms of *P. aeruginosa*, supporting its classification as an opportunistic pathogen capable of colonization and persistence across diverse environments [7,14]. The conservation of determinants associated with chronic biofilm formation underscores the ability of these isolates to survive under hospital-associated stress conditions and to drive prolonged outbreaks [17,57]. Conversely, heterogeneity in genes involved in initial adhesion and QS regulation may reflect divergent adaptive trajectories, consistent with the recognized adaptive plasticity of *P. aeruginosa* [60,61,62,63]. Variability in T3SS effector genes is clinically relevant, as these determinants have been associated with differences in cytotoxicity and disease severity [64,65]. Finally, the variable presence of genes related to iron regulation, siderophore biosynthesis, and heme uptake highlights the importance of iron metabolism as a key determinant of virulence and survival within hospital niches [66,67].

The predominance of ST111 and ST253 determined by WGS is epidemiologically relevant because these lineages can spread and persist in hospital settings, which favors the emergence of outbreaks. In particular, ST111 has been recognized as a high-risk clone with high therapeutic complexity and high clinical impact, described in several outbreaks of *P. aeruginosa* associated with carbapenemases, including VIM [33,68,69,70,71]. Similarly, ST253 stands out for its persistence and ability to prolong in different environments and its multidrug resistance profiles [72,73]. The study by Zurita et al., conducted in the city of Quito, like our study, determined that of 45 clinical isolates of *P. aeruginosa*, the most prevalent were ST253 and ST3750 which is closely related to the ST111 clonal complex, and concluded that these high-risk clones are contributing to the spread of multidrug resistance in Quito, Ecuador [74].

MALDI-TOF MS enabled the consistent identification of all outbreak isolates as *P. aeruginosa* with species-level scores, in agreement with previous studies highlighting the high accuracy and cost-effectiveness of this technology for routine clinical identification [29,75,76]. In addition, advances in Bruker analytical software (https://www.bruker.com/) have enabled the creation of dendrograms and PCA based on proteomic spectra, supporting the assessment of spectral similarity and enhancing its applicability in hospital surveillance contexts. Our results support the use of MALDI-TOF MS not only as an identification tool, but also as an operational surveillance layer capable of rapidly screening related isolates and guiding the prioritization of samples for genomic analysis. Such spectral analyses have been explored as tools to quickly identify high-risk clusters during outbreaks, although their discriminatory power depends on pre-analytical and analytical conditions, they provide valuable information on proteomic relatedness [26,77]. While MALDI-TOF MS does not replace high-resolution genomic typing or clonal inference, its use as a complementary approach offers comparative and exploratory value in outbreak investigations [30,32,78].

Although the proteomic proximity inferred by MALDI-TOF MS may partially reflect genomic relatedness determined by WGS, several studies have explored the degree of concordance between clustering patterns obtained using both approaches. In this study, predominant concordance between proteomic and genomic profiles was observed, in agreement with previous studies of nosocomial outbreaks. For example, Schlebusch et al. used tanglegram analysis to demonstrate partial overlap between WGS-based phylogeny and MALDI-TOF MS clustering in an outbreak of vancomycin-resistant enterococci (VRE) [31]. Similarly, Jürgen et al. reported that MALDI-TOF MS clusters largely corresponded to WGS-derived genotypic groupings in isolates from an outbreak involving *Serratia marcescens* and *Citrobacter freundii* [79]. Bar-Meir et al. also described compatible clustering patterns between MALDI-TOF MS and WGS during an outbreak of extended-spectrum β-lactamase-producing *Klebsiella pneumoniae* (ESBL-KP), emphasizing the value of MALDI-TOF MS as a useful and rapid tool for initial screening of the outbreak [32]. However, the discordance observed at the cluster level and the repositioning of three isolates in this study indicate that MALDI-TOF MS should not be interpreted as a substitute for WGS for clonal inference, but rather as a rapid screening and surveillance-oriented clustering tool that enables initial proteomic-level screening of potential outbreak clusters within minutes. In addition, it can provide early warnings of resistance, support real-time epidemiological surveillance, facilitate the processing of large numbers of samples in a short time at low cost, and its integration with genomic approaches is increasingly being explored [29,30,80,81].

Although the results in this study provide consistent evidence, it is important to recognize certain limitations that may influence interpretation. The genomic results of the resistome and virulome were obtained from assemblies generated exclusively from reads using the Oxford Nanopore technique, which generated variability in the completeness of the genomes. In this context, the heat maps should be interpreted with caution because they could underestimate the gene content, mainly in genomes with lower completeness. For this reason, priority was given to comparing the presence of genes rather than specific absences.

## 4. Materials and Methods

### 4.1. Initial Identification of Pseudomonas aeruginosa Isolates and Associated Clinical Data

One environmental strain and 38 clinical isolates of *P. aeruginosa* recovered from the ICU of Hospital IESS Quito Sur (Quito, Ecuador) were provided together with anonymized clinical records, supported by the corresponding ethical and institutional approval (protocol 2022-037M). Initial identification of *P. aeruginosa* was performed using phenotypic characteristics observed on MacConkey Agar and CHROMagar (Becton, Dickinson and Company, Franklin Lakes, NJ, USA), selective and differential media, which allow the identification of non-lactose-fermenting colonies and chromogenic differentiation based on the characteristic and classic blue-green pigmentation, respectively. The isolates were stored in cryovials at −20 °C until further analysis.

### 4.2. Antimicrobial Susceptibility Testing and VIM Carbapenemase Detection

Antimicrobial susceptibility testing was performed by broth microdilution assays using the BD Phoenix™ automated system (Becton Dickinson and Company, USA) with an antibiotic panel including amikacin, cefepime, ceftazidime, ciprofloxacin, gentamicin, meropenem, piperacillin-tazobactam, and colistin. Results were interpreted according to the breakpoints established by Clinical and Laboratory Standards Institute (CLSI) M100 guidelines [82] and classified as susceptible, intermediate, or resistant. In addition, the presence of VIM-type carbapenemases was assessed in respiratory sample isolates and blood cultures with the pneumonia and BCID2 panels, respectively, using the BioFire^®^ FilmArray^®^ 2.0 multiplex PCR system (BioMérieux, Craponne, France). Briefly, samples were prepared according to the manufacturer’s instructions, loaded into the cartridge, and processed with the fully automated assay, which detected *bla*_VIM_. Carbapenemases in urine samples were evaluated using the NG-TEST^®^ CARBA-5 lateral flow immunochromatographic assay (NG Biotech, Guipry-Messac, France), which directly detects the VIM enzyme. Briefly, the isolated colonies were homogenized in the extraction buffer, placed in the cassette, and the results were interpreted 15 min later based on the presence of the specific VIM and test control bands.

### 4.3. Determination of the Biofilm Formation Index (BFI)

The BFI was determined based on previous studies by Lucero-Mejía et al. and Atiencia-Carrera et al., with slight modifications [83,84]. Briefly, 200 µL of a bacterial suspension from each *P. aeruginosa* isolate was placed in 96-well microtiter plates at a final concentration of 1 × 10^7^ colony-forming units (CFU)/mL. The plates were incubated at 37 °C for 24 h. Absorbance was measured at 570 nm using the ELx808IU spectrophotometer (Bio Tek Instruments Inc., Winooski, VT, USA) to determine the OD_570nm_ of the cell culture (CC). Subsequently, phosphate-buffered saline (PBS) washes were performed to remove planktonic cells, the biofilm was resuspended in PBS, and absorbance was measured again to determine the OD_570nm_ of the biofilm (B). Each assay was performed in triplicate across three independent experiments, including blank or sterility controls (SC). The following formula was used to calculate the BFI for each isolate:

(1) $$BFI=\frac{B-SC}{CC-SC}$$

The level of biofilm formation for each isolate was classified into four categories according to the BFI value—none (<0.35), weak (0.35 to 0.69), moderate (0.70 to 1.09), and strong (≥1.10)—as reported in previous studies [51,83,85,86].

### 4.4. Whole Genome Sequencing (WGS)

For genomic characterization, 12 isolates representative of the outbreak were selected, capturing the greatest diversity. The selection was based on the inclusion of isolates belonging to different clusters established by MALDI-TOF MS, covering different points in time, and representing the variation in antimicrobial resistance profiles and types of origin, including the environmental isolate. Genomic DNA was extracted from each isolate using a Nucleic Acid Extraction Rapid kit (magnetic bread method) with the SSNP-A6 Automated Extraction System (Jiangsu Bioperfectus Technologies Co., Taizhou, China). Whole genome sequencing was performed using the Oxford Nanopore Technologies MinION platform (Oxford, UK), employing an R10.4.1 flow cell.

### 4.5. Bioinformatic Analysis

Basecalling was performed in real time during sequencing using Dorado (v7.6.8) integrated in MinKNOW (v24.11.10). Read quality was assessed using NanoPlot (v1.46.1) and SeqKit (v2.10.1), adapter trimming was conducted with Porechop (v0.2.4). De novo genome assembly was carried out using Flye (v2.9.5-b1801). Assembly polishing was performed using two rounds of Racon (v1.4.20), followed by consensus refinement with Medaka (v2.0.1). Assembly quality was evaluated using QUAST (v5.2.0), and genome completeness was assessed with BUSCO (v6.0.0). In addition, sequencing coverage was estimated by mapping reads to the final assembly using minimap2 (v2.28-r1209) and Samtools (v1.21). Taxonomic assignment was confirmed using Kraken2 (v2.1.3) and sequence types (ST) were assigned using Torsten Seemann’s mlst tool (v2.23.0), which queries MLST alleles via BLASTN (v2.15.0). Finally, genome annotation was performed with Bakta (v1.11.4) using the Bakta database (Bakta DB), and antimicrobial resistance determinants or resistome were identified using AMRFinderPlus (v3.12.8), and virulence-associated genes or virulome were inferred from annotated genomes through targeted gene searches using predefined gene lists and Bakta annotations (present, fragmented as pseudogene and absent). This bioinformatics workflow comprises widely used and validated tools, consistent with approaches applied in previous studies [66,67].

### 4.6. Proteomic Analysis Using MALDI-TOF MS

The identification of isolates was confirmed by MALDI-TOF MS with a MALDI Biotyper system (Bruker Daltonics, Bremen, Germany), according to the manufacturer’s instructions. Briefly, colonies from each isolate were transferred to individual wells of a polished steel target plate and coated with 1 µL of α-cyano-4-hydroxycinnamic acid (HCCA) matrix solution. Spectra were acquired by the equipment, and results were compared using the MBT Compass Explorer software (v3.7) and the BDAL MSP reference library. Proteomic analyses were performed using MALDI Biotyper Compass Explorer software (v4.1.100), starting with spectral preprocessing (mass adjustment, smoothing, baseline subtraction, normalization, and peak picking), followed by exploration of clustering patterns using PCA and the construction of dendrograms based on distances between spectral profiles, as previously described [78,87]. In addition, pseudo-gel visualizations were generated for the qualitative assessment of band patterns and peak consistency among isolates.

### 4.7. Comparison Between MALDI-TOF MS Dendrogram and Phylogeny by WGS

For the comparison of cluster groupings between proteomic and genomic profiles, a spectral similarity dendrogram was constructed using the MALDI Biotyper Compass Explorer software (v4.1.100), including only 12 selected and sequenced isolates. The genomic relationships among the same isolates were evaluated based on assembled genomes by aligning the core genome with Parsnp (v2.1.5), with PA_14 used as the reference genome. The phylogenetic tree was obtained in Newick format from the alignment of polymorphic sites (parsnp.snps.mblocks), and pairwise genomic distances (Hamming distance) were calculated. Tree visualization was performed using the Interactive Tree Of Life (iTOL) platform. Finally, a tanglegram was generated to compare cluster groupings obtained by the two methods.

### 4.8. Data Availability

Metadata were deposited in the NCBI database under Bioproject accession PRJNA1403182 and BioSample accessions (PA_02: SAMN54621036; PA_11: SAMN54621037; PA_14: SAMN54621038; PA_15: SAMN54621039; PA_21: SAMN54621040; PA_22: SAMN54621041; PA_23: SAMN54621042; PA_27: SAMN54621043; PA_28: SAMN54621044; PA_31: SAMN54621045; PA_32: SAMN54621046; PA_35: SAMN54621047).

### 4.9. Graphical Representations

Data visualization, including box-and-whisker plots and heat maps, was generated using GraphPad Prism (v8.0.1) [88]. Boxplots display the median and interquartile range, with whiskers indicating the minimum and maximum values.

### 4.10. Ethical Statements

This study included only bacteria isolated from human biological samples obtained as part of routine microbiological diagnosis. In addition, only anonymized clinical metadata were used. The study protocol was reviewed and approved by the Comité de Ética de Investigación en Seres Humanos (CEISH) of the Universidad San Francisco de Quito (USFQ) (approval code 2022-037M; approval date: 6 April 2023).

## 5. Conclusions

In summary, this hospital outbreak was characterized by multidrug-resistant *Pseudomonas aeruginosa* isolates, with a predominance of carbapenem resistance, ST111 and ST253 linages, and a consistent ability to form moderate to strong biofilms, including isolates recovered from abiotic surfaces such as sinks and central venous catheters. These features reinforce the clinical risk posed by this pathogen in critical care units and support its high potential for environmental persistence and sustained transmission during prolonged outbreaks.

The integrated molecular characterization performed in this study highlights the value of MALDI-TOF MS and MALDI Biotyper Compass Explorer software not only for rapid species identification but also as a screening and surveillance-oriented clustering tool that provides early epidemiological insights. When combined with whole genome sequencing, MALDI-TOF MS contributes complementary information that enhances outbreak characterization through the integration of phylogenetic relationships, resistome, and virulome profiles.

The predominant concordance observed between MALDI-TOF MS-based clustering determined by MALDI Biotyper Compass Explorer software and WGS phylogeny supports the combined use of proteomic and genomic approaches as an effective strategy for outbreak investigation. Together, these tools enable timely decision-making, improve epidemiological surveillance, and support the implementation of targeted infection control measures to mitigate the impact of hospital outbreaks caused by *P. aeruginosa*.

## Figures and Tables

**Table 1:** Clinical and epidemiological characteristics of *Pseudomonas aeruginosa* isolates.

Isolate ID	Clinical Specimen	*P. aeruginosa*-Associated Diagnosis	Date of Isolation	Patient Age (Years)	Patient Sex
PA_01	Tracheal aspirate	HAP	3 March 2022	84	M
PA_02	Tracheal aspirate	HAP	8 February 2022	54	M
PA_03	BAL	HAP	17 March 2022	68	M
PA_04	Blood culture	Sepsis	27 March 2022	56	M
PA_05	Tracheal aspirate	HAP	23 March 2022	76	M
PA_06	Tracheal aspirate	HAP	30 March 2022	76	M
PA_07	Blood culture	Urosepsis	27 March 2022	56	M
PA_08	Urine	UTI	12 April 2022	34	M
PA_09	Sputum	HAP	9 May 2022	68	M
PA_10	Urine	UTI	10 May 2022	68	M
PA_11	Tracheal aspirate	HAP	14 May 2022	49	M
PA_12	Tracheal aspirate	Septic shock	30 May 2022	49	M
PA_13	Tracheal aspirate	HAP	28 May 2022	51	F
PA_14	Blood culture	Bacteremia	6 June 2022	65	M
PA_15	Tracheal aspirate	HAP	12 June 2022	65	M
PA_16	Blood culture	Septic shock	18 June 2022	49	M
PA_17	Tracheal aspirate	HAP	14 February 2022	54	M
PA_18	Blood culture	Bacteremia	16 June 2022	49	M
PA_19	Tracheal aspirate	HAP	1 July 2022	75	M
PA_20	Tracheal aspirate	HAP	27 September 2021	78	F
PA_21	Blood culture	Bacteremia	17 September 2022	26	M
PA_22	Blood culture	Septic shock	26 September 2022	48	F
PA_23	Urine	UTI	20 September 2022	48	F
PA_24	Tracheal aspirate	HAP	19 September 2022	48	F
PA_25	Blood culture	Septic shock	18 September 2022	48	F
PA_26	Blood culture	Bacteremia	21 September 2022	55	M
PA_27	Tracheal aspirate	HAP	22 September 2022	60	M
PA_28	Tracheal aspirate	HAP	17 September 2022	67	M
PA_29	Urine	UTI	19 September 2022	85	M
PA_30	Blood culture	Bacteremia	28 October 2022	39	M
PA_31	Blood culture	Bacteremia	18 July 2022	79	M
PA_32	Blood culture	Septic shock	6 November 2022	74	M
PA_33	Blood culture	Bacteremia	8 November 2022	72	M
PA_34	CVC	Septic shock	8 November 2022	72	M
PA_36	Tracheal aspirate	HAP	16 January 2023	1	F
PA_37	Tracheal aspirate	HAP	7 February 2023	81	M
PA_38	Tracheal aspirate	HAP	18 February 2023	68	F
PA_39	Tracheal aspirate	HAP	24 February 2023	81	M

BAL: Bronchoalveolar lavage; CVC: Central venous catheter; M: Male; F: Female; HAP: Hospital-acquired pneumonia; UTI: Urinary tract infection.

**Table 2:** Antimicrobial susceptibility profiles of *P. aeruginosa* isolates.

Isolate ID	AMK	FEP	CAZ	CIP	GEN	MEM	TZP	Colistin MIC (mg/L)	VIM Determination
PA_01	I	R	R	R	S	R	I	1	ND
PA_02	S	R	R	R	R	R	I	2	*bla*_VIM_
PA_03	S	S	S	S	S	S	S	<1	ND
PA_04	S	S	S	R	R	R	S	2	ND
PA_05	S	R	R	I	I	R	I	2	ND
PA_06	S	R	R	R	R	R	R	>4	ND
PA_07	S	S	S	R	R	R	S	2	ND
PA_08	S	R	S	R	R	R	R	1	ND
PA_09	S	R	R	R	R	R	I	<1	*bla*_VIM_
PA_10	S	R	R	R	R	R	I	<1	VIM
PA_11	S	R	R	R	R	R	R	2	*bla*_VIM_
PA_12	S	R	R	R	R	R	R	2	ND
PA_13	S	R	R	R	S	R	R	1	*bla*_VIM_
PA_14	S	S	S	S	S	S	S	1	ND
PA_15	S	R	R	R	R	R	R	1	ND
PA_16	S	R	R	R	R	R	R	1	ND
PA_17	S	R	R	R	R	R	I	2	ND
PA_18	S	R	R	R	I	R	R	1	ND
PA_19	S	R	R	R	R	R	I	1	*bla*_VIM_
PA_20	S	R	R	R	R	R	I	1	*bla*_VIM_
PA_21	S	S	S	S	S	S	S	<1	ND
PA_22	R	R	R	R	S	R	I	<1	*bla*_VIM_
PA_23	S	R	R	R	S	S	S	1	VIM
PA_24	R	R	R	R	S	R	I	2	*bla*_VIM_
PA_25	R	R	R	R	S	R	I	2	*bla*_VIM_
PA_26	R	R	R	R	S	R	R	≤1	*bla*_VIM_
PA_27	R	R	R	R	S	R	I	≤1	*bla*_VIM_
PA_28	S	R	R	R	R	R	R	1	ND
PA_29	R	R	R	R	S	R	I	≤1	VIM
PA_30	S	S	S	S	S	S	S	1	ND
PA_31	S	S	S	S	S	S	S	1	ND
PA_32	S	R	R	R	R	R	R	1	*bla*_VIM_
PA_33	S	R	R	R	R	R	R	1	*bla*_VIM_
PA_34	S	R	R	R	R	R	R	1	*bla*_VIM_
PA_36	S	R	R	R	S	R	R	1	*bla*_VIM_
PA_37	S	R	R	R	R	R	R	1	*bla*_VIM_
PA_38	S	R	R	R	R	R	R	1	*bla*_VIM_
PA_39	S	R	R	R	R	R	R	1	*bla*_VIM_

R: resistant; I: intermediate; S: susceptible; MIC: minimum inhibitory concentration; AMK: amikacin; FEP: cefepime; CAZ: ceftazidime; CIP: ciprofloxacin; GEN: gentamicin; MEM: meropenem; TZP: piperacillin-tazobactam; *bla*_VIM_: gene encoding Verona integron-encoded metallo-β-lactamase; VIM: Verona integron-encoded metallo-β-lactamase; ND: evaluated and not detected.

## Data Availability

The metadata were deposited in NCBI under Bioproject PRJNA1403182 and BioSample accessions (PA_02: SAMN54621036; PA_11: SAMN54621037; PA_14: SAMN54621038; PA_15: SAMN54621039; PA_21: SAMN54621040; PA_22: SAMN54621041; PA_23: SAMN54621042; PA_27: SAMN54621043; PA_28: SAMN54621044; PA_31: SAMN54621045; PA_32: SAMN54621046; PA_35: SAMN54621047).

## Supplementary Materials

The following supporting information can be downloaded at: https://www.mdpi.com/article/10.3390/antibiotics15030257/s1, Table S1. Biofilm formation index (BFI) of *P. aeruginosa* isolates; Table S2. Assembly metrics, mapping, and BUSCO completeness for sequenced *Pseudomonas aeruginosa* outbreak isolates; Table S3. Resistome gene list and genomic information for sequenced isolates; Table S4. Virulome gene list and genomic information for sequenced isolates; Table S5. MALDI-TOF MS-based identification of *P. aeruginosa* outbreak isolates. Table S6. Matrix of pairwise SNP distances derived from the core-genome alignment.

## Abbreviations

The following abbreviations are used in this manuscript:

AMK	Amikacin
BAL	Bronchoalveolar lavage
BFI	Biofilm formation index
*bla*_VIM_	Gene encoding Verona integron-encoded metallo-β-lactamase
CAZ	Ceftazidime
CIP	Ciprofloxacin
CLSI	Clinical and Laboratory Standards Institute
CRPA	Carbapenem-resistant *Pseudomonas aeruginosa*
CVC	Central venous catheter
ESBL-KP	Extended-spectrum β-lactamase-producing *Klebsiella pneumoniae*
FEP	Cefepime
GEN	Gentamicin
HAIs	Healthcare-associated infections
HAP	Hospital-acquired pneumonia
HCCA	α-cyano-4-hydroxycinnamic acid
I	Intermediate
ICU	Intensive care unit
iTOL	Interactive Tree Of Life
LPS	Lipopolysaccharide
MALDI-TOF MS	Matrix-assisted laser desorption/ionization time-of-flight mass spectrometry
MBLs	Metallo-β-lactamases
MEM	Meropenem
MIC	Minimum inhibitory concentration
PCA	Principal component analysis
PBS	Phosphate-buffered saline
QS	Quorum sensing
R	Resistant
S	Susceptible
SNPs	Single-nucleotide polymorphisms
ST	Sequence type
T3SS	Type III secretion system
T6SS	Type VI secretion system
TZP	Piperacillin-tazobactam
UTI	Urinary tract infection
VIM	Verona integron-encoded metallo-β-lactamase
VRE	Vancomycin-resistant enterococci
WHO	World Health Organization
WGS	Whole genome sequencing
