# Supplementary material for: Integrated Phenotypic, Proteomic (MALDI-TOF MS), and Genomic (WGS) Investigation of a Prolonged Hospital Outbreak of Pseudomonas aeruginosa with High Biofilm-Forming Capacity
Source: Antibiotics (Basel). 2026 Mar 2;15(3):257. doi: 10.3390/antibiotics15030257 (PMC13023606; doi:10.3390/antibiotics15030257)
Supplement: Supplementary file 1 [file antibiotics-15-00257-s001.zip › Supplementary Table S1,S5.pdf]

Supplementary Table S1. Biofilm formation index (BFI) of *P. aeruginosa* isolates.

Isolate ID	BFI (mean ± SD)	Biofilm category
PA_01	0.86 ± 0.06	moderate
PA_02	1.12 ± 0.08	strong
PA_03	1.11 ± 0.05	strong
PA_04	0.83 ± 0.09	moderate
PA_05	0.95 ± 0.06	moderate
PA_06	1.11 ± 0.04	strong
PA_07	0.98 ± 0.03	moderate
PA_08	1.05 ± 0.08	moderate
PA_09	1.10 ± 0.05	strong
PA_10	1.10 ± 0.07	strong
PA_11	1.10 ± 0.04	strong
PA_12	0.90 ± 0.08	moderate
PA_13	0.89 ± 0.03	moderate
PA_14	0.99 ± 0.07	moderate
PA_15	0.90 ± 0.07	moderate
PA_16	1.02 ± 0.05	moderate
PA_17	1.00 ± 0.08	moderate
PA_18	0.95 ± 0.05	moderate
PA_19	1.11 ± 0.06	strong
PA_20	0.95 ± 0.04	moderate
PA_21	0.93 ± 0.09	moderate
PA_22	0.99 ± 0.08	moderate
PA_23	1.07 ± 0.06	moderate
PA_24	1.10 ± 0.08	strong
PA_25	0.92 ± 0.07	moderate
PA_26	0.97 ± 0.04	moderate
PA_27	1.10 ± 0.05	strong
PA_28	0.82 ± 0.04	moderate
PA_29	0.99 ± 0.09	moderate
PA_30	0.97 ± 0.07	moderate
PA_31	0.99 ± 0.06	moderate
PA_32	1.00 ± 0.03	moderate
PA_33	1.00 ± 0.06	moderate
PA_34	1.10 ± 0.06	strong
PA_35	1.11 ± 0.09	strong
PA_36	0.96 ± 0.06	moderate
PA_37	1.04 ± 0.08	moderate
PA_38	0.83 ± 0.02	moderate
PA_39	0.93 ± 0.07	moderate

BFI value: none (<0.35), weak (0.35 to 0.69), moderate (0.70 to 1.09), and strong (≥1.10).

Supplementary Table S5. MALDI-TOF MS-based identification of *P. aeruginosa* outbreak isolates.

Isolate ID	Identification (top hit)	Score value	Consistency category
PA_01	*Pseudomonas aeruginosa* DSM 1117 DSM	2.19	A
PA_02	*Pseudomonas aeruginosa* ATCC 27853 THL	2.45	A
PA_03	*Pseudomonas aeruginosa* 8147 2 CHB	2.03	B
PA_04	*Pseudomonas aeruginosa* ATCC 27853 THL	2.28	A
PA_05	*Pseudomonas aeruginosa* DSM 1117 DSM	2.25	A
PA_06	*Pseudomonas aeruginosa* ATCC 27853 THL	2.31	A
PA_07	*Pseudomonas aeruginosa* ATCC 27853 THL	2.41	A
PA_08	*Pseudomonas aeruginosa* LMG 8029 LMG	2.07	A
PA_09	*Pseudomonas aeruginosa* ATCC 27853 THL	2.18	A
PA_10	*Pseudomonas aeruginosa* ATCC 27853 THL	2.21	A
PA_11	*Pseudomonas aeruginosa* ATCC 27853 THL	2.16	A
PA_12	*Pseudomonas aeruginosa* ATCC 27853 THL	2.08	A
PA_13	*Pseudomonas aeruginosa* ATCC 27853 THL	2.09	B
PA_14	*Pseudomonas aeruginosa* DSM 1117 DSM	2.48	A
PA_15	*Pseudomonas aeruginosa* ATCC 27853 THL	2.29	A
PA_16	*Pseudomonas aeruginosa* ATCC 27853 THL	2.35	A
PA_17	*Pseudomonas aeruginosa* ATCC 27853 THL	2.39	A
PA_18	*Pseudomonas aeruginosa* ATCC 27853 THL	2.28	C
PA_19	*Pseudomonas aeruginosa* ATCC 27853 THL	2.32	A
PA_20	*Pseudomonas aeruginosa* ATCC 27853 THL	2.25	A
PA_21	*Pseudomonas aeruginosa* ATCC 27853 THL	2.32	A
PA_22	*Pseudomonas aeruginosa* ATCC 27853 THL	2.35	A
PA_23	*Pseudomonas aeruginosa* DSM 1117 DSM	2.28	A
PA_24	*Pseudomonas aeruginosa* ATCC 27853 THL	2.38	A
PA_25	*Pseudomonas aeruginosa* DSM 1117 DSM	2.38	A
PA_26	*Pseudomonas aeruginosa* ATCC 27853 THL	2.13	A
PA_27	*Pseudomonas aeruginosa* 19955 1 CHB	2.16	A
PA_28	*Pseudomonas aeruginosa* 8147 2 CHB	2.20	A
PA_29	*Pseudomonas aeruginosa* DSM 1117 DSM	2.36	A
PA_30	*Pseudomonas aeruginosa* ATCC 27853 THL	2.31	A
PA_31	*Pseudomonas aeruginosa* DSM 1117 DSM	2.42	A
PA_32	*Pseudomonas aeruginosa* ATCC 27853 THL	2.27	A
PA_33	*Pseudomonas aeruginosa* ATCC 27853 THL	2.39	A
PA_34	*Pseudomonas aeruginosa* ATCC 27853 THL	2.26	A
PA_35	*Pseudomonas aeruginosa* 19955 1 CHB	2.32	A
PA_36	*Pseudomonas aeruginosa* ATCC 27853 THL	2.31	A
PA_37	*Pseudomonas aeruginosa* DSM 1117 DSM	2.32	A
PA_38	*Pseudomonas aeruginosa* ATCC 27853 THL	2.20	A
PA_39	*Pseudomonas aeruginosa* ATCC 27853 THL	2.35	A

Score value interpretation: 2.300 – 3.000, highly probable species identification; 2.000 – 2.299, secure genus identification and probable species identification. Consistency categories interpretation: A, species consistency; B, genus consistency; C, neither species nor genus consistency.
